# The first reported case of *Blaptica dubia* cockroach allergy

**DOI:** 10.1186/s13223-021-00618-2

**Published:** 2021-11-02

**Authors:** Hannah Wangberg, Jun Mendoza, Robert Gomez, Christopher Coop, Andrew White, Katharine Woessner

**Affiliations:** 1 Scripps Clinic Department of Allergy, Asthma, and Immunology, 3811 Valley Centre Dr. S99, San Diego, CA 92130 USA; 2 Wilford Hall Ambulatory Surgical Center, San Antonio, TX USA

**Keywords:** Allergy, Asthma, Allergic rhinitis, Cockroach, *Blaptica dubia*, Dubia cockroach, *Periplaneta americana*, American cockroach, *Blattella germanica*, German cockroach

## Abstract

**Background:**

*Periplaneta americana* and *Blattella germanica* cockroaches are widespread, and risk of sensitization increases in urban environments where these roaches thrive as household pests. There are no prior reports of *Blaptica dubia* cockroach allergy, though human exposure to *B. dubia* is increasing through commercial breeding as feeder insects.

**Case presentation:**

A 50-year-old *B. dubia* cockroach breeder presented with progressively worsening upper and lower respiratory symptoms in recent years. Symptoms were worse with exposure to her B. dubia roach colony. Skin prick testing (SPT) to *B. dubia* cast skin, internal organs, and feces was performed in both the subject and a human control. Testing for *P. americana* and *B. germanica* sensitization was also performed in the subject*. *SDS–Polyacrylamide gel electrophoresis (PAGE), immunoblots, and enzyme-linked immunosorbent assays (ELISA) studies were performed using the subject and control serums to explore for specific IgE binding to *B. dubia* as well as *P. americana. *Our results showed SPT was positive to *B. dubia* internal organs in the subject and negative in the control. In the subject, SPT was negative to *P. americana* though intradermal (ID) testing was positive and serum specific IgE (sIgE) testing was negative to *B. germanica*. Immunoblotting of the subject's serum to *B. dubia* internal organ extract showed several distinct bands of IgE binding at 47 kilodaltons (kD), 68 kD, 74 kD, 83 kD, and 118 kD. The strongest band was at 118 kD on *B. dubia* immunoblotting, which was absent in *P. americana* on SDS-PAGE. ELISA studies showed an increased IgE response to both *B. dubia* and *P. americana* in the subject versus the control.

**Conclusions:**

This case confirmed the first reported allergy to *B. dubia* cockroaches. There may be cross-reactivity between *B. dubia* and *P. americana*, though our case suggests SPT and sIgE testing using *P. americana* and *B. germanica* extract has potential to miss a *B. dubia* cockroach allergy. This allergy is likely underreported, and further study is needed to explore the natural history of *B. dubia* cockroach allergy.

## Background

Cockroaches are widely abundant insects worldwide, though only a small fraction of the approximately 4500 species in existence are adapted to human habitats [1]. Of the domiciliated cockroach species, the American Cockroach (*Periplaneta americana*) and German Cockroach (*Blattella germanica*) are the most pervasive pests [1, 2]. Exposure to both *P. americana* and *B. germanica* have demonstrated potential to evoke IgE-mediated allergic sensitization in humans [2, 3]. It is estimated that 15–60% of atopic individuals are sensitized to one of these cockroaches, and the risk of sensitization increases significantly among atopic individuals with heightened cockroach exposure as may occur in urban dewllings [2, 4–7] Among sensitized individuals, *P. americana* or *B. germanica* cockroach exposure may exacerbate symptoms associated with both allergic rhinitis and asthma [4, 8, 9].

To date, 12 allergens have been associated with *B. germanica* and 13 with *P. americana* as listed on the World Health Organization and International Union of Immunological Societies (WHO/IUIS) Allergen Nomenclature Database [3]. While not confirmed on the WHO/IUIS database, other domiciliated cockroach species throughout the world and are hypothesized to have varying degrees of cross-reactivity to *B. germanica* and *P. americana* [1].

The Orange-Spotted Cockroach/Argentinian Wood Roach (*Blaptica dubia)* is a tropical cockroach species that is not endemic to human dwellings, though human exposure to *B. dubia* is increasing with their widespread commercial breeding as feeder insects [3, 10]. Feeder insects are bred with the purpose of being used as food for other animals, typically reptiles. While no reports of *B. dubia* allergy exist in medical literature, websites for *B. dubia* breeders have described the potential to develop allergic symptoms with *B. dubia* exposure [11, 12]. In this report, we present the first confirmed case of *B. dubia* allergy in a Dubia cockroach breeder.

## Case presentation

The subject was a 50-year-old female who presented with progressively worsening symptoms of dyspnea, wheezing, cough, and nasal congestion. She had been breeding *B. dubia* cockroaches in her home for commercial sale to pet shops for the past several years. Initially, she had no symptoms with exposure to the *B. dubia* cockroaches, though recently she had noticed marked worsening of her upper and lower airway symptoms with exposure to her *B. dubia* roach colony. She reported that once a *B. dubia* cockroach ran across her arm and she broke out in an urticarial rash where it had touched her skin. She did not reside in an inner city dwelling nor was there evidence of a cockroach infestation in her home.

### Skin testing

Skin testing was performed to multiple aeroallergens including *P. americana* using commercial extract *(*GreerLaboratories GB26A03) and to non-standardized *B. dubia* cockroach cast skin, internal organs, and feces particles. Each *B. dubia* sample for skin prick testing (SPT) was mixed with saline and SPT conducted in both the subject and a non-cockroach allergic atopic human control (Table 1)*.* Serum specific IgE (sIgE) testing was performed to *B. germanica* in the subject as this extract was not available at the time of skin testing*.*

**Table 1:** Skin testing results

Prick	Wheal	Flare	Interpretation
SPT to common Aeroallergens			
Histamine	10	40	–
Glycerine 50%	0	0	–
** 7 Grass Mix**	**20**	**40**	**Positive**
** Bermuda Grass**	**25**	**40**	**Positive**
** Johnson Grass**	**20**	**40**	**Positive**
** S. Calif Weed Mix B**	**10**	**25**	**Positive**
** West 10 Tree Mix**	**10**	**25**	**Positive**
** S. Calif 6 Tree Mix**	**6**	**22**	**Positive**
Mold Mix #1	0	0	Negative
Mold Mix #2	0	0	Negative
Aspergillus	0	0	Negative
Candida	0	0	Negative
Cockroach (*P. americana,* GB26A03)	0	0	Negative
Dog Epithelia	0	0	Negative
Feathers Mix	0	0	Negative
Horse Epithelia	0	0	Negative
Cat Hair	0	0	Negative
**Dust Mite**	**9**	**15**	**Positive**
SPT to B. dubia^*^in subject			
Histamine (subject)	10	23	–
Glycerin 50% (subject)	0	0	–
* B. dubia* cast skin (subject)	0	0	Negative
*** B. dubia* internal organs (subject)**	**6**	**9**	**Positive**
* B. dubia* feces (subject)	0	0	Negative
*SPT to B. dubia in control*			
Histamine (control)	6	25	–
Glycerin 50% (control)	0	0	–
* B. dubia* cast skin (control)	0	0	Negative
* B. dubia* internal organs (control)	0	0	Negative
* B. dubia* feces (control)	0	0	Negative
Intradermal testing in subject			
Histamine	15	35	–
Glycerin 50%	3	0	–
** Cockroach (*P. americana* GB26A03)**	**10**	**30**	**Positive**

Bolded items indicate skin tests that were interpreted as positive

*B. dubia* *Blaptica dubia* cockroaches, *SPT* Skin prick testing

^*^SPT to *B. dubia* internal organs, cast skin, and feces was not performed on the same day of SPT to common aeroallergens in the subject

### Cockroach protein extraction for in vitro studies

Cockroach protein extracts were made in lab for in vitro studies. Briefly, 2.5 g of material isolated from *B. dubia* cast skins, internal organs, and *P. americana* whole body extract (WBE) were separately incubated with 25 ml of extraction buffer for 18 h at 4 ℃. Samples were centrifuged at 2000 revolutions per minute (rpm) for 20 min. The supernatant was sequentially filtered with 8, 3, 1.2, 0.8, and 0.45 micron filter membranes. Each filtrate was dialyzed using a 3.5 kD pore membrane and run against distilled water for 24 h with a water exchange at 3, 6, 16, and 20 h. The dialysates were frozen at −20 ℃ and the frozen dialysates were lyophilized, and 1 mg of this lyophilized product was tested for protein concentration (µg protein/mg extract) using a Modified Micro-Protein Lowry method.

### Enzyme-linked immunosorbent assay (ELISA)

The subject’s serum and a non-atopic human control’s serum were added to ELISA plate wells coated with cockroach protein extracts and incubated overnight at room temperature. These plate wells had been coated with 100 µg of the different cockroach protein extracts in 100µl of carbonate coating buffer and then incubated overnight at 4 °C (set up in triplicates) and then washed. This was blocked with 200 µL 3% bovine serum albumin (BSA)/tris-buffered saline (TBS) for 1 h at 37 °C and then removed. Samples were then added to ELISA plate wells, incubated overnight at room temperature, then washed.

100 µl of monoclonal mouse IgG to human immunoglobulin E (anti-IgE) (1:300 dilution in 3% BSA/TBS) conjugated to alkaline phosphatase was then added followed by 100 µL of p-Nitrophenyl Phosphate (pNPP) alkaline phosphatase substrate. An average of triplicate optical densities (OD) were obtained. OD ratios (subject/negative control) were used to determine the subject’s “IgE response” to various cockroach protein extracts.

### Sodium dodecyl sulfate polyacrylamide gel electrophoresis (SDS-PAGE) and immunoblot

**Fig. 1:**
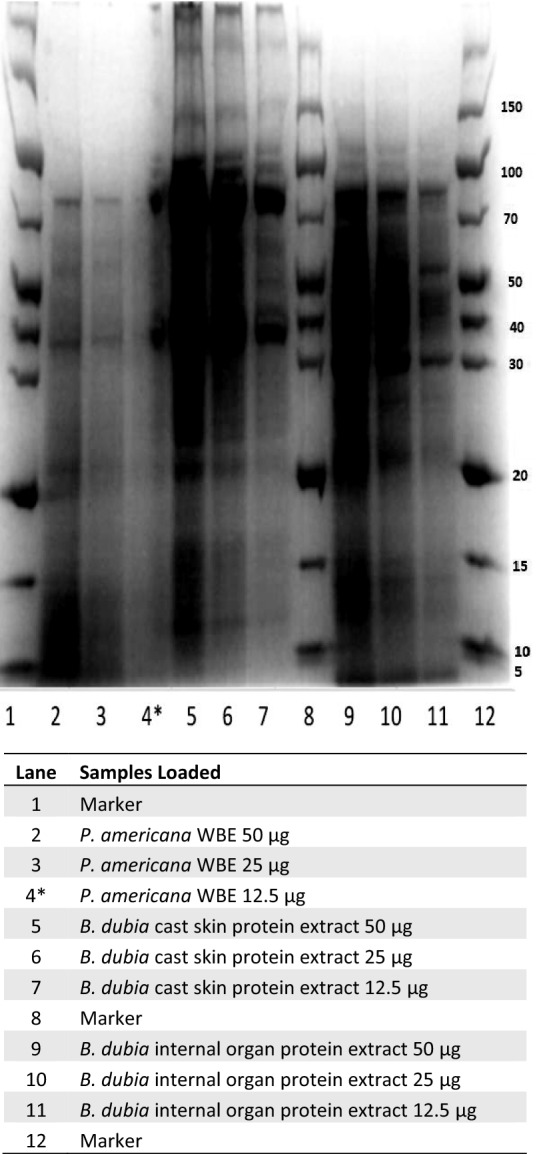
SDS–polyacrylamide gel electrophoresis of *P. americana* whole body protein extract, *B. dubia* cast skin protein extract, and *B. dubia* internal organs protein extract. *Some spill-over from lane 5 into lane 4

The SDS-PAGE ran for 1.5 h at 130 V with 50 µg, 25 µg, and 12.5 µg of each denatured protein extract (*B. dubia* cast skins, *B. dubia* internal organs, and *P. americana* WBE) as shown in Fig. 1. A marker/ladder was also loaded, as well as a denature buffer only for blank wells. The gel was stained with Coomassie Blue R250, de-stained, and then photographed and the concentration with the best resolution of protein bands was chosen for downstream immunoblot.

**Fig. 2:**
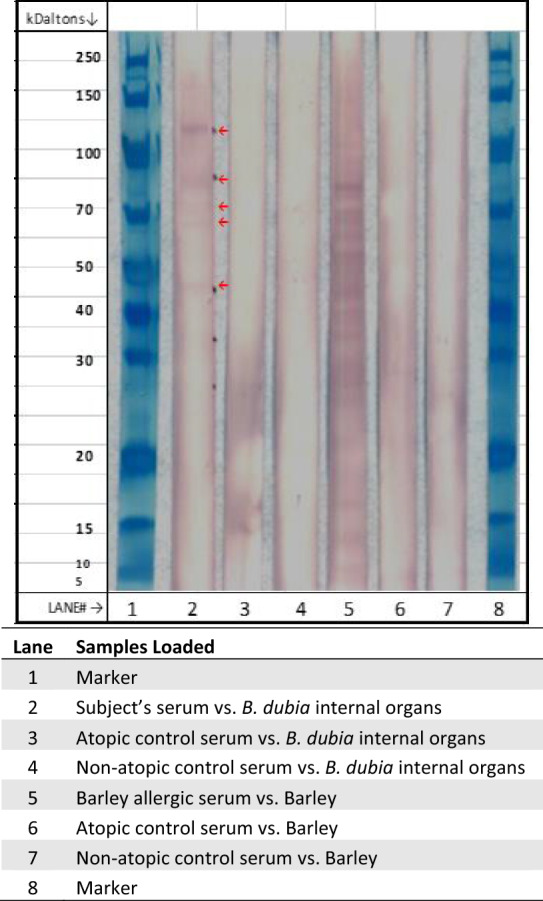
Immunoblot results. Red arrows (lane 2) reflect the subject’s serum demonstrating IgE binding to *B. dubia* internal organ protein extract. Bands were identified at approximately 118 kD, 83 kD, 74 kD, 68 kD, and 47 kD. Positive controls (lane 5) and negative controls (lane 3, 4, 6, 7) performed as expected

The immunoblot was performed to *B. dubia* internal organ protein extract. 25 µg of *B. dubia* internal organ denatured protein extract was loaded in three separate lanes of gel (Fig. 2). The gel ran for 1.5 h at 130 V and then transferred onto a 0.45 micron nitrocellulose membrane for 2 h at 36 V. The membrane was removed and cut into strips, placed into reaction troughs, and washed. Strips were blocked for 1 hour at room temperature with 5% non-fat dry milk (NFDM) then aspirated. 0.3 ml of each sample in 2.7 ml of 5% NFDM were added to reaction troughs, incubated overnight at room temperature, aspirated, and then washed. 3 ml of a 1:250 monoclonal, mouse immunoglobulin-G (IgG) to anti-human immunoglobulin-E (IgE) (AP conjugated) in 5% NFDM was then added and incubated for 4 h at room temperature then washed. 3ml of development buffer was added to each trough and observed for color development, then washed with 5ml of deionized H2O for 10 minutes with one change of water at 5 min (Fig. 2).

## Results

SPT and intradermal (ID) skin testing results are shown in Table 1. Results were notable for a negative SPT in the subject to *P. americana* though ID testing was positive. *B. dubia* SPT was positive to internal organs in the subject and negative in the control (Table 1). sIgE testing of the subject’s serum was negative to *B. germanica* (<0.35 kU/L). The cockroach protein extractions for in vitro studies yielded 728 μg protein/mg weight for *P. americana* WBE, *B. dubia* cast skin yielded 744 μg protein/mg weight, and *B. dubia* internal organs yielded 1000 μg protein/mg weight. SDS-PAGE results for the two *B. dubia* extracts and *P. americana* WBE are shown in Fig. 1. Immunoblotting of the subject’s serum with *B. dubia* internal organ extract detected five bands of IgE binding at 47 kD, 68 kD, 74 kD, 83 kD, and a strong band at 118 kD (Fig. 2). Positive and negative control reagents performed as expected (Fig. 2). The 118 kD denatured protein was present in *B. dubia* cast skin and internal organ extracts but not *P. americana* WBE on SDS-PAGE (Fig. 1). ELISA of the subject’s serum versus *B. dubia* internal organs, cast skin, and *P. americana* WBE found IgE OD detected at a ratio of 7.2×, 8.6×, and 6.8× greater than the negative control, respectively.

## Discussion and conclusions

Cockroaches produce potent allergens, though the vast majority of human exposure to cockroaches are to domiciliated house pests such as *P. americana* and *B. germanica*. This case represents the first confirmed *B. dubia* cockroach allergy in a cockroach breeder with evidence of IgE-mediated sensitization to multiple *B. dubia* allergens. This sensitization almost certainly contributed to the subject’s allergic rhinitis and asthma that developed through her occupational exposure to *B. dubia*.

Intradermal and in vitro studies suggest the subject was sensitized to *P. americana* but not *B. germanica* cockroach. The false negative SPT we observed to commercial *P. americana* extract was likely related to the variability of protein concentrations within commercially available cockroach extracts [13]. We suspect the subject also had false negative SPT to *B. dubia* cast skin, as in-vitro studies suggested the subject was sensitized to *B. dubia* cast skin and her clinical history of contact urticaria when a roach ran across her arm also suggest sensitization to external cockroach allergens. We hypothesize this probable false negative SPT to *B. dubia* cast skin likely occurred because the saline mixed with cast skin for SPT did not elicit significant amount of protein. This was a limitation of our investigation, as were unable to utilize the *B. dubia* cockroach extracts made in lab for the subject’s SPT. The subject’s IgE sensitization to *P. americana* may be due to unbeknownst prior exposure and sensitization to this cockroach species. Is also possible that cross-reactivity between *B. dubia* and *P. americana* resulted in positive *P. americana* testing. It is noteworthy that the most robust IgE binding on immunoblot to *B. dubia* internal organ extract occurred at the high molecular weight 118 kD allergen. By comparison, this 118 kD protein was absent in *P. americana* WBE on SDS-PAGE. These findings may suggest that at least one of the several *B. dubia* allergens identified were unrelated to potential cross-reactivity with *P. americana* cockroach.

## Conclusion

We confirmed the first known allergy to *B. dubia* cockroaches with evidence of IgE sensitization to *B. dubia* and symptoms of allergic rhinitis, asthma, and contact urticaria with *B. dubia* exposure. This allergy is likely underreported, and allergists should be aware of the potential for allergic sensitization to *B. dubia* roaches. Our case suggests SPT or in vitro testing for *B. dubia* cockroach allergy using *P. americana* or *B. germanica* extracts has potential to miss a *B. dubia* allergy. Should a patient be suspected to have a *B. dubia* allergy, cockroach avoidance would likely be the optimal approach. However, many individuals with *B. dubia* exposure rely on cockroach breeding as a source of income [11], and alternatives to strict avoidance may be desired. In such instances, personal protective equipment should be encouraged to mitigate the risk of sensitization and morbidity associated with cockroach allergy. Further research is needed to explore *B. dubia* cockroach allergy.

## Data Availability

The datasets during and/or analyzed during the current study available from the corresponding author on reasonable request.

## Abbreviations

*B*. *dubia*: Blaptica dubia
*B*. *germanica*: Blattella germanica
BSA: Bovine serum albumin
ELISA: Enzyme-linked immunosorbent assay
ID: Intradermal
kD: KiloDalton
OD: Optical densities
pNPP: p-Nitrophenyl Phosphate
*P*. *americana*: *Periplaneta* *americana*
RPM: Revolutions per minute
sIgE: Serum specific immunoglobulin-E
SDS-PAGE: Sodium dodecyl sulfate polyacrylamide gel electrophoresis
SPT: Skin prick test
TBS: Tris-buffered saline
WBE: Whole body extract
WHO/IUIS: World Health Organization and International Union of Immunological Societies
